# Target position reproducibility in left‐breast irradiation with deep inspiration breath‐hold using multiple optical surface control points

**DOI:** 10.1002/acm2.12321

**Published:** 2018-05-08

**Authors:** Aurora Fassi, Giovanni B. Ivaldi, Paola Tabarelli de Fatis, Marco Liotta, Ilaria Meaglia, Patrizia Porcu, Lea Regolo, Marco Riboldi, Guido Baroni

**Affiliations:** ^1^ Dipartimento di Elettronica, Informazione e Bioingegneria Politecnico di Milano Milano Italy; ^2^ Department of Radiation Oncology Istituti Clinici Scientifici Maugeri Pavia Italy; ^3^ Division of Medical Physics Istituti Clinici Scientifici Maugeri Pavia Italy; ^4^ Division of Breast Surgery Istituti Clinici Scientifici Maugeri Pavia Italy; ^5^ Bioengineering Unit Clinical Division CNAO Foundation Pavia Italy

**Keywords:** left‐breast DIBH radiotherapy, optical tracking system, surface fiducials, target reproducibility

## Abstract

The aim of this study was to investigate the use of 3D optical localization of multiple surface control points for deep inspiration breath‐hold (DIBH) guidance in left‐breast radiotherapy treatments. Ten left‐breast cancer patients underwent whole‐breast DIBH radiotherapy controlled by the Real‐time Position Management (RPM) system. The reproducibility of the tumor bed (i.e., target) was assessed by the position of implanted clips, acquired through in‐room kV imaging. Six to eight passive fiducials were positioned on the patients' thoraco‐abdominal surface and localized intrafractionally by means of an infrared 3D optical tracking system. The point‐based registration between treatment and planning fiducials coordinates was applied to estimate the interfraction variations in patients' breathing baseline and to improve target reproducibility. The RPM‐based DIBH control resulted in a 3D error in target reproducibility of 5.8 ± 3.4 mm (median value ± interquartile range) across all patients. The reproducibility errors proved correlated with the interfraction baseline variations, which reached 7.7 mm for the single patient. The contribution of surface fiducials registration allowed a statistically significant reduction (*p *< 0.05) in target localization errors, measuring 3.4 ± 1.7 mm in 3D. The 3D optical monitoring of multiple surface control points may help to optimize the use of the RPM system for improving target reproducibility in left‐breast DIBH irradiation, providing insights on breathing baseline variations and increasing the robustness of external surrogates for DIBH guidance.

## 1 INTRODUCTION

Deep inspiration breath‐hold (DIBH) is a widely adopted clinical protocol for the radiotherapy treatment of left‐sided breast cancer patients after breast‐conserving surgery.^1^ By reducing lung density and increasing the distance between the heart and the chest wall, the DIBH technique allows the improvement in heart and lung sparing.^2^ Several studies report the benefit of DIBH strategy in decreasing cardio‐pulmonary exposure dose compared to free‐breathing (FB) treatments,^3^ without compromising target coverage.^4^ This can potentially contribute to a reduction in radiation‐induced cardiac diseases and pulmonary complications, which are considered one of the major factors affecting the overall survival in left‐breast patients after postoperative radiotherapy.^5^ Despite the clear advantages of DIBH treatments, there are issues associated to the possible inaccuracy in dose delivery, due to intra and interfraction uncertainties in target position among repeated DIBHs.^6^, ^7^ The assessment and selection of the most appropriate technology for DIBH monitoring is crucial to ensure an effective radiation treatment.

Image‐guided radiation therapy (IGRT) techniques based on fluoroscopy or portal images have been successfully applied to verify the level of deep inspiration in left‐breast patients, obtaining a setup variability within 2 mm.^8^ However, these IGRT methods pose concerns about the additional patient exposure to non‐therapeutic radiation dose. The most common non‐ionizing techniques for respiratory motion monitoring in DIBH treatments are the spirometry‐based Active Breathing Coordinator (ABC) system (Elekta, Stockholm, Sweden) and the video‐based Real‐time Position Management (RPM) system (Varian Medical Systems, Inc., Palo Alto, CA, USA).^1^ The ABC device measures through spirometric techniques the volume of air inspired and expired by the patient, which is used to control the DIBH level.^9^ In the RPM optical tracking system, DIBHs are monitored by an external surface surrogate, represented by the vertical displacement of a fiducial box usually placed on the patients' xiphoid process and acquired with a single infrared (IR) camera.^10^ However, neither of these techniques use target breast position to gate treatment, which may result in less accurate dose delivery. We have previously demonstrated that spirometry‐based control does not always guarantee a stable and reproducible position of the external breast surface in left‐breast DIBH radiotherapy.^11^ Other studies also found that the RPM system alone may not be an adequate surrogate for the definition of the DIBH level in left‐breast treatments.^6^, ^7^, ^12^ The reason is that the RPM technique consists of the 2D monitoring of a single surface point, with a relative measurement of the breathing baseline.^6^

Several works have recently investigated the use of 3D surface imaging, such as the AlignRT system (VisionRT Ltd, London, UK), to improve the reproducibility and stability of DIBH in left‐breast radiotherapy.^6^, ^13^, ^14^, ^15^, ^16^ This approach employs optical surface detection techniques to reconstruct the 3D external topology of the breast, which is rigidly registered to the reference planning surface to obtain the breathing surrogate for DIBH monitoring. Three‐dimensional surface imaging resulted in a mean setup uncertainty of 2 mm^15^, ^16^ and proved to be more correlated with the target position compared to the RPM system.^6^ An alternative approach is represented by the use of multiple passive fiducials placed on different surface points and reconstructed in 3D using IR optical tracking systems.^11^, ^17^ The fiducial‐based approach allows the fast 3D tracking of reliable control points attached to the skin surface. Multiple fiducials tracking has already been proposed to guide DIBH treatments for extracranial tumors with inter‐breath‐hold reproducibility below 3 mm,^18^, ^19^ but to our knowledge no clinical application has been implemented yet.

The aim of this study was to investigate the use of 3D optical localization of multiple surface fiducials for DIBH control in left‐breast radiotherapy treatments. DIBH reproducibility is assessed in terms of external surface positioning and internal target localization error, using the position during fractions of implanted radiopaque clips identified with kV imaging. The performance of the proposed method in controlling patients' breath‐hold level is compared to the clinical RPM system. The assessment of the RPM reproducibility for DIBH control has already been investigated in previous studies, based on the deviation of the chest wall (CW) position obtained from X ray images.^6^, ^7^, ^12^ The limitation of these works is the use of a 2D surrogate as target position, which does not allow a full 3D displacement analysis. In our study, the 3D position of clips implanted in the tumor bed at the time of surgery was used as reference and the contribution of multiple surface control points was investigated to compensate for absolute breathing baseline variations and to provide a more reliable surrogate, using corresponding point‐based registration.

## 2 MATERIALS AND METHODS

### 2.1 Patient dataset

The study included 10 left‐sided breast cancer patients treated with whole‐breast tangential radiotherapy under DIBH technique, controlled with the RPM system. For these patients, the DIBH treatment was selected rather than the traditional tangential FB treatment to satisfy the dosimetric heart constraint (i.e., the volume of heart receiving more than 5 Gy had to be less than 5%). Before CT simulation, each patient underwent a training session to select the appropriate DIBH level. The RPM fiducial box was positioned on the patients' upper abdomen, using skin marking to reproduce its positioning during subsequent simulation and treatment fractions. The DIBH level was set around the 90% of the maximum vertical displacement reached by the RPM fiducial box during training relatively to the patients' breathing baseline, obtained by averaging the box position in FB. The gating window was set to ±2.5 mm around the DIBH level. The selected DIBH level and gating window were applied to perform DIBH maneuvers during simulation and treatment phases. Audio respiratory coaching was used to guide the patients' breathing trace to reach and maintain the correct DIBH level.

As shown in Table 1, patients had 3 to 7 titanium clips secured by the surgeons in the excision cavity wall during lumpectomy, to better identify in CT images the tumor bed for the electron boost irradiation. The implanted radiopaque clips were used to assess the reproducibility of the target (i.e., the tumor bed) during simulation and treatment DIBHs. The evaluation of the external surface reproducibility was based on multiple skin landmarks (nevi or scars) of the patients' thoraco‐abdominal surface. Six to eight surface points were identified during the training session for each patient (Table 1), considering at least one landmark on the left‐breast. A picture of the selected skin landmarks was taken to allow the recognition of the corresponding surface points during the subsequent simulation and treatment fractions.

**Table 1:** Patient dataset acquired for the study

Patient	Number of implanted clips	Number of surface fiducials	Number of analyzed treatment fractions	Number of analyzed DIBH maneuvers
P1	3	7	10	48
P2	5	8	10	46
P3	6	6	15	60
P4	6	7	13	64
P5	6	8	11	76
P6	3	7	15	59
P7	6	8	12	58
P8	6	8	12	47
P9	6	8	11	43
P10	7	8	12	72

**Figure 1:**
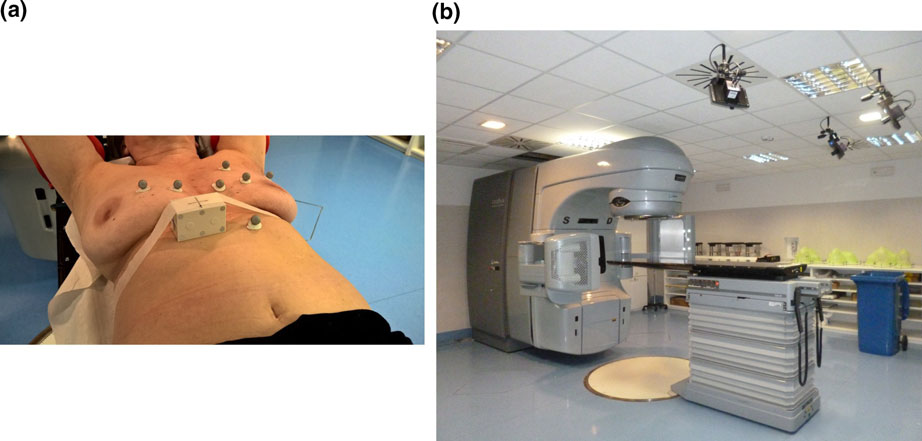
Panel (a) depicts the thoraco‐abdominal surface of a breast cancer patient, with multiple fiducials placed on the selected skin landmarks and with the RPM fiducial box fixed in the upper‐abdomen region. Panel (b) shows the three IR cameras of the optical tracking system installed in the treatment room.

At the time of CT simulation, radiopaque fiducials (BTS Bioengineering, Garbagnate Milanese, Italy) were placed on the identified skin landmarks, as well as the RPM fiducial box [Fig. 1(a)]. A FB scan and an RPM‐controlled DIBH scan were acquired for each patient using the GE Light Speed RT 16 CT scanner (GE Healthcare, Milwaukee, WI, USA). The voxel size of the acquired CT scans was 0.94 × 0.94 × 2.5 mm^3^. The FB CT dataset was used to set the isocenter position for the daily patient setup. The DIBH CT scan, acquired in a single DIBH maneuver, was used for treatment planning (Philips Pinnacle v9.0, Philips Radiation Oncology Systems, Fitchburg, WI, USA) and for daily portal imaging verification. The whole breast was considered as the clinical target volume (CTV), while the planning target volume (PTV) was defined by adding a 10 mm margin to the CTV.

Patients were treated with hypo‐fractioned whole‐breast radiotherapy with the Clinac iX accelerator (Varian Medical Systems, Inc., Palo Alto, CA, USA). The prescribed dose was 2.25 Gy/fraction, for a total of 45 Gy delivered in 20 daily fractions over 4 weeks, plus a weekly tumor bed boost of 1.25 Gy. At the beginning of each treatment fraction, passive fiducials with an IR‐reflective coating (BTS Bioengineering, Garbagnate Milanese, Italy) were positioned on the corresponding surface landmarks. The optical tracking system SMART DX‐100 (BTS Bioengineering, Garbagnate Milanese, Italy) was used in a multi‐camera configuration to record the 3D trajectories of the passive fiducials during the whole treatment fraction. As depicted in Fig. 1(b), the optical tracking system is composed by three IR cameras rigidly fixed to the ceiling of the treatment room and provides the 3D reconstruction of fiducials in the isocentric reference system with millimetric accuracy at 100 Hz frame rate. As described in details in previous works,^20^, ^21^ the isocentric calibration of the optical tracking system is based on the BrainLab (BrainLAB AG, Feldkirchen, Germany) phantom, which is fitted with a known geometric configuration of passive fiducials. The phantom is manually aligned to the isocentric room lasers to find the mapping between the optical cameras reference system and the isocentric room reference frame.

For patient setup, an initial laser‐based alignment was performed on skin tattoos in FB. Patient positioning was verified both in FB and DIBH by acquiring daily MV electronic portal images at the treatment gantry angles. According to the adopted clinical protocol, if setup errors were higher than 3 mm for the single spatial dimension, setup corrections were applied to the treatment couch and a portal verification was repeated. Two to four opposed tangential photon beams were delivered in consecutive DIBH maneuvers. Beam energies of 6–15 MV were used, by applying high‐dose rates of 500–600 MU/min to limit the delivery time up to 20 s per beam. For 10 to 15 treatment fractions (Table 1), kV imaging was acquired after tangential beams to localize internal clip positions in the 3D space for offline analysis. Two X ray projections were captured with the On‐Board Imaging (OBI) system at 0° and 315°. An angular difference of 45° was chosen between the two projections to limit the time associated to the X ray source‐panel rotation, thus allowing the acquisition of both kV images within the same DIBH maneuver. For each patient, only the treatment fractions in which kV imaging was acquired were analyzed for the study. Depending on the number of portal verifications and delivered tangential beams, 4 to 7 DIBH maneuvers were performed per fraction.

### 2.2 Data analysis

The reference planned position of the internal clips and external surface fiducials were obtained from the planning CT volumes. Radiopaque clips were manually segmented by a clinician in the DIBH CT scan, obtaining the so‐called *clip DIBH CT model (c_BH*_*CT*_ *)*. Surface fiducials were automatically extracted from both FB and DIBH CT images,^22^ deriving the *fiducial FB CT model (f_FB*_*CT*_ *)* and the *fiducial DIBH CT model (f_BH*_*CT*_ *)*, respectively. To evaluate the reproducibility of the DIBH maneuvers, clips and fiducials planned positions were compared to the corresponding coordinates acquired during the treatment phase. For each analyzed fraction, the information on clips positions was available only for the last DIBH maneuver associated to kV imaging. The 3D clips coordinates were reconstructed from the two acquired kV projections through stereo‐triangulation techniques. Clips coordinates in 2D were manually identified on each kV image and 3D localization was derived using the direct linear transformation algorithm, obtaining the so‐called *clipDIBH kV position (c_BH*_*kV*_ *)*.

The 3D coordinates of surface fiducials were continuously acquired by the optical system for the entire treatment course. For each treatment fraction, the following variables were computed for all fiducials (due to the non‐Gaussian distribution of fiducial trajectories, a non‐parametric statistic was applied to compute the variables):

- *fiducial FB treatment position (f_FB*_*treat*_ *)*, defined as the median fiducial position acquired in FB at the beginning of the treatment fraction, namely during the 10 breathing cycles preceding portal acquisition;
- *fiducial DIBH treatment position (f_BH*_*treat*_ *)*, defined as the median fiducial position acquired during the different DIBH maneuvers performed in the considered treatment fraction, when the RPM fiducial box was within the predefined DIBH window;
- *fiducial DIBH kV position (f_BH*_*kV*_ *)*, defined as the median fiducial position acquired during the DIBH maneuver associated to kV imaging in the considered treatment fraction.

**Figure 2:**
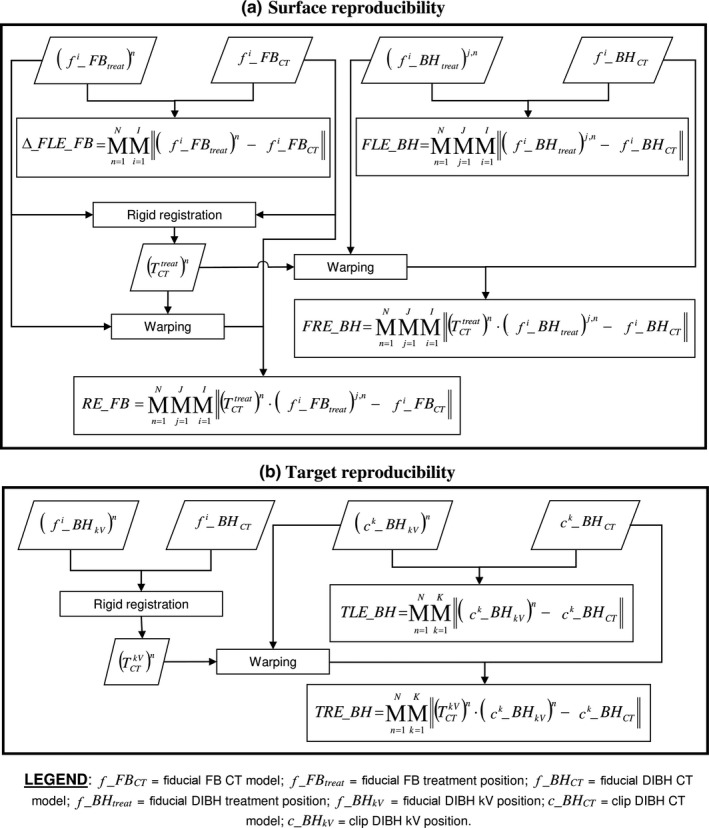
Definition of the indices used to assess surface (a) and target (b) reproducibility. The symbol M denotes the median operation. *I* represents the number of fiducials placed on the patients' surface, while *K* is the number of implanted clips. The number of analyzed treatment fractions is defined as *N*, whereas *J* represents the number of DIBHs performed in each fraction.

Specific indices were defined to assess the reproducibility of the external surface and internal target position during DIBH radiotherapy (Fig. 2). The so‐called *Localization* indices were used to evaluate DIBH reproducibility under the control of the RPM system, which defines the gating window based on a relative 2D FB baseline. The *Registration* indices were used to assess the improvement in DIBH reproducibility using multiple fiducials placed on the patients' thoraco‐abdominal surface. In all cases, indices were calculated by averaging over all the available fiducials, to minimize the uncertainties due to possible fiducials misplacement among different fractions.

The absolute 3D localization of the fiducials was exploited to measure the absolute FB baseline and to correct possible baseline variations that can occur between planning and treatment phases. External surface reproducibility was assessed with the following indices [Fig. 2(a)]:

- *FB baseline variation (Δ_FLE_FB)*, which was defined as the Euclidean distance between *f_FB*_*CT*_ and *f_FB*_*treat*_, mediated over all fiducials and over all analyzed treatment fractions; ‐ *fiducial localization error (FLE_BH)*, evaluating the surface reproducibility under RPM‐based DIBH control. *FLE_BH* was computed as the Euclidean distance between *f_BH*_*CT*_ and *f_BH*_*treat*_, mediated over all fiducials and over all DIBHs performed in the analyzed treatment fractions;
- *fiducial registration error (FRE_BH)*, estimating the surface reproducibility after a fiducial‐based FB baseline correction. The transformation matrix$T_{\mathrm{CT}}^{\mathrm{treat}}$, defining FB baseline variation from planning to treatment, was obtained by rigidly registering *f_FB*_*treat*_ to *f_FB*_*CT*_ *. FRE_BH* was computed as the Euclidean distance between *f_BH*_*CT*_ and *f_BH*_*treat*_, after applying the baseline correction matrix $T_{\mathrm{CT}}^{\mathrm{treat}}$, mediated over all fiducials and over all DIBHs performed in the analyzed treatment fractions. The *residual errors* (*RE_FB*) of the rigid registration applied for baseline correction provided information on the interfraction accuracy in the manual repositioning of surface fiducials. The *RE_FB* was defined as the Euclidean distance between *f_FB*_*CT*_ and *f_FB*_*treat*_, after applying the baseline correction matrix $T_{\mathrm{CT}}^{\mathrm{treat}}$, mediated over all fiducials and over all analyzed treatment fractions.

The indices regarding internal target reproducibility were the following [Fig. 2(b)]:

- *target localization error (TLE_BH)*, evaluating the target reproducibility under RPM‐based DIBH control. The *TLE_BH* was computed as the Euclidean distance between *c_BH*_*CT*_ and *c_BH*_*kV,*_ mediated over all clips and over all analyzed treatment fractions;
- *target registration error (TRE_BH)*, estimating the target reproducibility under a fiducial‐based DIBH control. The transformation matrix $T_{\mathrm{CT}}^{\mathrm{kV}}$, defining the fiducial‐based correction to be applied to clip coordinates, is obtained by rigidly registering *f_BH*_*kV*_ to *f_BH*_*CT*_ *. TRE_BH* was computed as the Euclidean distance between *c_BH*_*CT*_ and *c_BH*_*kV*_, after applying the fiducial‐based correction matrix $T_{\mathrm{CT}}^{\mathrm{kV}}$, mediated over all clips and over all analyzed treatment fractions.

The applied method for 3D clip reconstruction assumes that no motion occurs between the acquisition of the two kV projections. To verify this condition, we assessed the stability of surface fiducials positions during kV imaging for each treatment fraction. The *kV stability* index was computed as the 5th–95th percentile range of each single fiducial coordinate acquired during kV maneuver, then mediated over all fiducials and over all spatial directions. The absence of clip migration during the treatment course was also verified using the Euclidean distance between pairs of clips (inter‐clip distance). The *clip migration* parameter was defined as the median difference between the corresponding inter‐clip distances measured from *c_BH*_*CT*_ and from *c_BH*_*kV*_ *,* mediated over all clips. Finally, the correlation between the internal target and the fiducials (*internal‐external correlation*) was estimated by computing for each spatial direction the Spearman's correlation coefficients between the *TLE_BH* of each treatment fraction and the corresponding translation parameters of the correction matrix$T_{\mathrm{CT}}^{\mathrm{kV}}$.

## 3 RESULTS

The resulting number of DIBH maneuvers analyzed per patient ranged from 43 to 76, as depicted in Table 1. Table 2 shows the surface reproducibility results, expressed as median value ± interquartile range (IQR). *Δ_FLE_FB* was higher than 3.2 mm for all patients, with a maximum value of 7.7 mm. For 6 over 10 patients, *FLE* exceeded 5 mm, ranging from 4.0 to 8.8 mm. A statistically significant correlation was found between *Δ_FLE_FB* and *FLE_BH* (Spearman's correlation coefficients = 0.75, *p *< 0.05). As depicted in Table 2, the fiducial‐based contribution for baseline correction allowed reducing surface reproducibility errors under 5 mm, with *FRE_BH* values ranging from 2.9 to 4.9 mm. A significant improvement was recorded from *FLE_BH* to *FRE_BH* for all patients (Wilcoxon rank test, *p *< 0.05) except for patient P9. The *RE_FB* for fiducial‐based rigid registration were lower than 3 mm for all patients (Table 2).

**Table 2:** Surface reproducibility results

Patient	Δ_FLE_FB (mm)	FLE_BH (mm)	FRE_BH (mm)	RE_FB (mm)
P1	4.7 ± 2.4	4.0 ± 1.9	3.2 ± 0.6	2.1 ± 1.5
P2	4.3 ± 2.6	6.4 ± 1.8	4.7 ± 1.6	2.6 ± 1.7
P3	5.8 ± 3.8	7.1 ± 1.9	3.2 ± 1.0	2.0 ± 1.1
P4	7.1 ± 3.8	6.0 ± 1.7	4.2 ± 1.6	2.8 ± 1.6
P5	7.0 ± 3.8	6.9 ± 1.0	2.9 ± 0.6	1.5 ± 0.9
P6	4.2 ± 2.2	4.4 ± 1.4	3.4 ± 1.3	1.7 ± 1.5
P7	3.2 ± 2.3	4.4 ± 1.4	3.2 ± 0.5	2.4 ± 1.2
P8	3.8 ± 2.6	4.9 ± 2.0	3.8 ± 1.4	1.6 ± 1.2
P9	6.0 ± 3.4	6.5 ± 3.9	4.9 ± 0.8	2.2 ± 1.9
P10	7.7 ± 3.3	8.8 ± 2.4	4.6 ± 0.8	2.9 ± 1.4
Median	5.3 ± 3.9	5.9 ± 2.8	3.6 ± 1.6	2.1 ± 1.6

Table 3 summarizes the results related to target reproducibility. The 3D clip reconstruction was considered reliable for all patients' fractions, since the *kV stability* index was below 3 mm. An upward trend of the *clip migration* parameter was recorded for all patients during the treatment course, but the maximum value did not exceed 3.5 mm for the single fraction. The results of *kV stability* and *clip migration* indices, mediated over all treatment fractions, are reported in Table 3 for each patient. Target reproducibility under the RPM‐based control proved significantly worse than under the fiducial‐based control (Wilcoxon rank test, *p *< 0.05). Values for *TLE_BH* up to 8.7 mm were recorded (patient P5), whereas *TRE_BH* values did not exceed 4.2 mm (Table 3).

**Table 3:** Target reproducibility results

Patient	kV stability (mm)	Clip migration (mm)	TLE_BH (mm)	TRE_BH (mm)
P1	0.8 ± 0.6	2.2 ± 1.4	4.7 ± 1.9	1.9 ± 1.3
P2	0.9 ± 0.9	1.9 ± 2.2	3.5 ± 3.1	2.6 ± 0.7
P3	1.2 ± 1.5	1.0 ± 1.1	8.4 ± 2.4	4.2 ± 1.1
P4	0.5 ± 0.4	2.0 ± 2.8	5.7 ± 4.3	3.8 ± 1.1
P5	0.7 ± 0.8	1.6 ± 2.5	8.7 ± 2.7	3.5 ± 1.0
P6	1.0 ± 1.0	1.0 ± 0.9	4.4 ± 1.2	2.9 ± 1.5
P7	0.8 ± 0.6	1.0 ± 1.4	7.1 ± 2.0	4.1 ± 1.4
P8	0.7 ± 0.8	1.3 ± 1.0	4.2 ± 1.8	3.0 ± 0.5
P9	0.7 ± 0.8	0.4 ± 0.5	5.8 ± 2.3	3.7 ± 3.2
P10	1.3 ± 0.7	1.8 ± 1.9	6.6 ± 2.1	3.8 ± 1.7
Median	0.9 ± 0.8	1.2 ± 1.5	5.8 ± 3.4	3.4 ± 1.7

**Figure 3:**
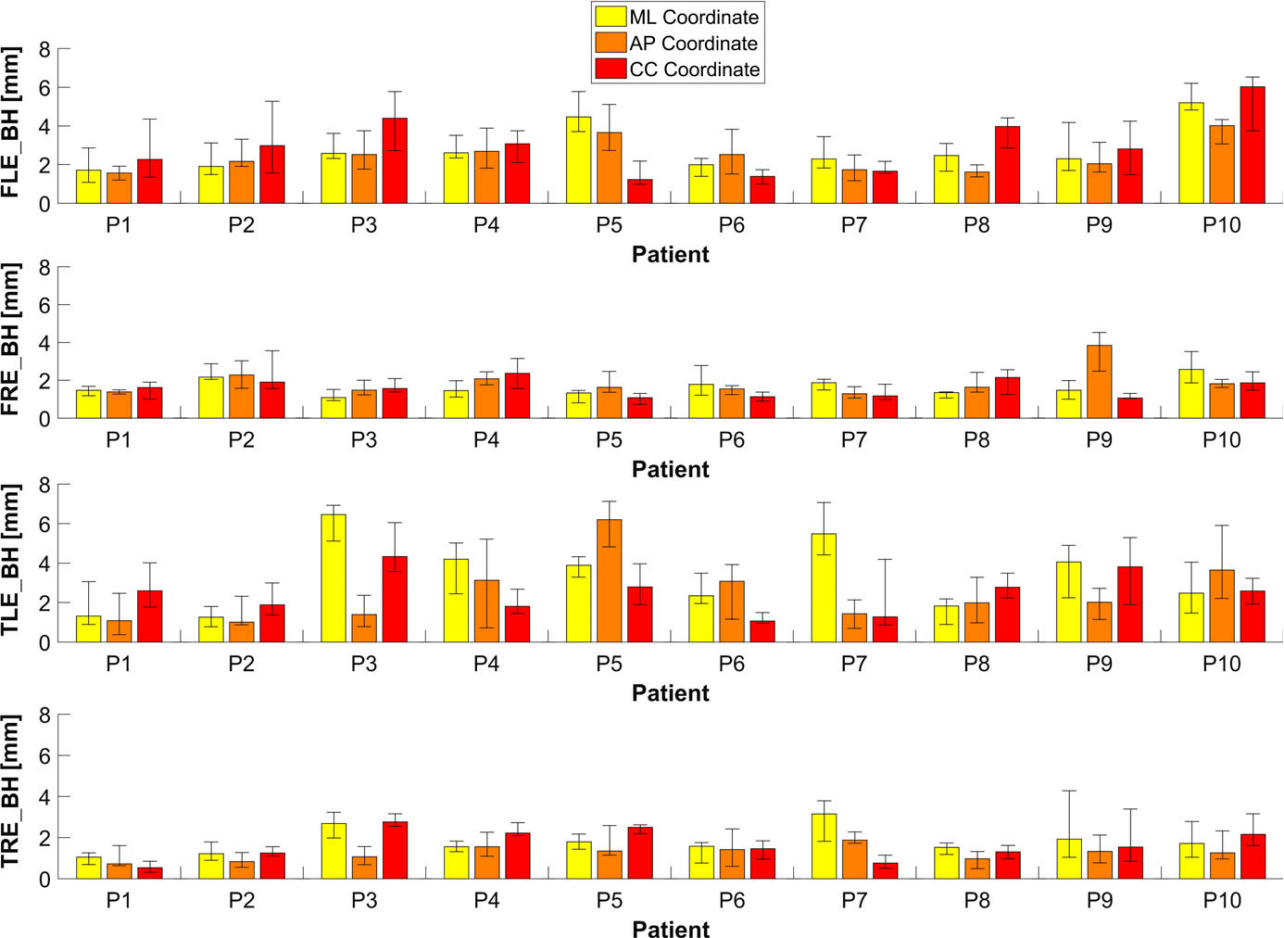
Absolute errors (median ± IQR) for surface and target reproducibility estimated along medio‐lateral (ML), antero‐posterior (AP) and cranio‐caudal (CC) directions.

**Figure 4:**
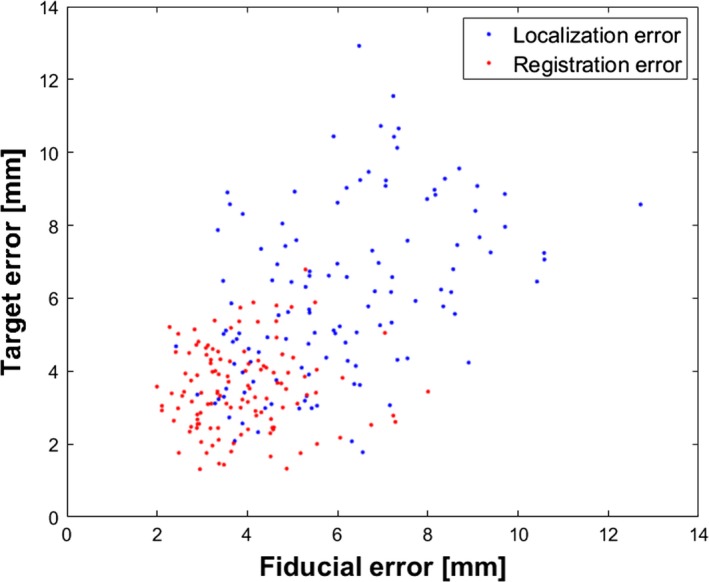
Median values of FLE_BH vs TLE_BH (blue dots) and FRE_BH vs TRE_BH (red plots) estimated for all patients and treatment fractions.

Figure 3 shows the surface and target reproducibility errors as a function of the spatial direction. *FLE_BH* values mediated over all patients measured 2.5, 2.5, and 2.7 mm for the medio‐lateral (ML), antero‐posterior (AP) and cranio‐caudal (CC) coordinates, respectively, with errors up to 6.0 mm for the single spatial direction (patient P10). *FRE_BH* did not exceed 3.9 mm for the single direction, with median values of 1.5, 1.7 and 1.5 mm for ML, AP and CC coordinates. Concerning target reproducibility, a *TLE_BH* up to 6.5 mm were recorded for patient P3, whereas *TRE_BH* values were lower than 3.2 mm for each spatial direction. For ML, AP and CC coordinates, respectively, median *TLE_BH* measured 3.1, 2.2, 2.4 mm, whereas *TRE* values were reduced to 1.7, 1.3 and 1.7 mm. A significant *internal‐external correlation* (*p *< 0.05) was found for all patients along AP and CC coordinates, with a median value of the Spearman's coefficients of 0.73 and 0.86, respectively. *FLE_BH*and *TLE_BH* were found to be significantly correlated (Spearman correlation coefficient *ρ *= 0.47, *p* < 0.05), whereas no correlation was found for *FRE_BH* and *TRE_BH* (Spearman correlation coefficient *ρ *= 0.04, *p* = 0.64) (Fig. 4).

## 4 DISCUSSION

Respiratory‐gating with DIBH can potentially reduce heart and pulmonary dose for left‐sided breast cancer patients. However, the efficacy of DIBH treatments strongly depends on target position reproducibility during breath‐holds, thus stressing the need to assess the adequacy of the applied respiratory monitoring system. The setup accuracy and DIBH reproducibility in left‐breast cancer radiotherapy is a clinically relevant and widely investigated topic.^6^, ^7^, ^11^, ^12^, ^13^, ^14^, ^15^, ^16^ Previous studies analyzed only the reproducibility of the patients' external surface, without any reference to the true target positioning.^13^, ^15^ In recent reports, target reproducibility was determined indirectly using CW excursion estimated on 2D X‐ray images.^6^, ^7^, ^12^, ^16^ In this study, DIBH reproducibility was evaluated considering both the patients' thoraco‐abdominal surface and the true target position. In particular, target localization was based directly on the 3D reconstruction of implanted clips and included the quantification of interfraction clip migration (Table 3).

A previous work demonstrated the lack of correlation between the clinical RPM system and CW position during left‐breast DIBH irradiation, concluding that the only use of RPM as gating surrogate may not be sufficient to ensure accurate DIBH treatment delivery.^6^ The inadequacy of the RPM‐based surrogate for the definition of the DIBH level was also assessed in Skyttä et al.^12^ and Lutz et al.,^3^ reporting occasional large errors in CW position up to 16.3 mm. Our study confirmed the inherent problem of the 2D RPM system, that is, the relative estimation of the patients' breathing baseline, which does not allow taking into account interfraction baseline shifts. We quantitatively assessed the variation in patients' breathing baseline under the RPM‐based DIBH control, which measured 5.3 ± 3.9 mm as mediated over all patients (Table 2). The median reproducibility errors obtained with the RPM‐based DIBH control was 5.9 mm for surface position (Table 2) and 5.8 mm for target position (Table 3). For 6 over 10 patients, the localization errors exceeded 5 mm, which was the selected RPM gating window. In our study, the RPM block was placed on the patients' abdomen, midway between the xyphoid process and the umbilicus, and a 5 mm gating window was selected, as recommended by the RPM manufacturer and in recent works.^23^, ^24^ The positioning of the fiducial block on the sternum or breast can result in improved DIBH reproducibility,^12^ but concerns are associated to the lateral block tilt and surface dose effects.^25^ A reduced gating window can also contribute to increase targeting accuracy, but there is the evidence that large errors in CW position can occur despite a small gating window.^7^ Moreover, a reduced gating window will potentially increase treatment difficulty and duration, due to a possible decrease in the duty cycle.

Different techniques have been proposed to improve DIBH reproducibility in left‐breast treatment controlled with the RPM system. In Skyttä et al.,^12^ a correction of the height of the RPM gating window was implemented based on the lateral kV setup images, resulting in a significant reduction in CW positional errors. Non‐invasive 3D surface imaging has also been proposed as a more reliable guidance technique for left‐breast DIBH treatments.^6^, ^13^, ^14^, ^15^, ^16^ The AlignRT gating surrogate is represented by the real‐time positioning offsets obtained from the rigid‐body transformation between the real surface of interest and the planned surface. A good correlation was found by Rong et al. between this surrogate and the CW excursion, obtaining an average intrafraction CW offset lower than 2.5 mm.^6^ However, the computational cost of surface acquisition and registration algorithms may limit the frame rate of DIBH monitoring. Depending on the size and resolution of AlignRT surface models, Rong et al. obtained a frame rate ranging from 0.5 to 1.6 Hz,^6^ which could not be sufficient to prevent relevant consequences in case of sudden large patient movements, especially if reduced margins and increased dose are required. The frame rate can be increased by reducing the surface region‐of‐interest, but surface fitting algorithms may result in inadequate setup accuracy in case of insufficient surface topology information.^26^

We investigated an alternative approach for left‐breast DIBH monitoring based on multiple surface fiducials localized by means of an optical tracking system. The proposed method is based on the point‐based registration between the fiducials positions acquired intrafractionally during FB and the corresponding planned FB CT coordinates. This approach can be applied to detect baseline shifts, which represent setup errors at FB, and to correct the height of the RPM gating window. The roto‐translational parameters obtained from fiducials registration can also be used as a more reliable surrogate for the DIBH monitoring of patients' breast position, without the need of the RPM system. A good correlation was found between the registration parameters and the target localization errors. The median reproducibility errors obtained with the fiducial‐based DIBH control on the analyzed patient dataset was 3.8 and 3.5 mm for surface and target position, respectively (Table 2 and 3). The obtained results are comparable to the reproducibility performance of the AlignRT method,^6^, ^13^, ^14^, ^15^, ^16^ whereas a significant improvement was recorded with respect to the RPM system. The benefits of the fiducial‐based technique are related to the real‐time performance, allowing a DIBH monitoring frequency up to 100 Hz, and to the reliable registration results, due to the use of corresponding surface points rather than non‐corresponding surface contours.

Possible concerns regarding the clinical application of the proposed fiducial‐based approach can be related to the time required for surface fiducials positioning and to the interfraction variability in fiducials repositioning on skin landmarks. In this study, the fiducials were positioned on the patients' surface before entering the treatment room, with no impact on the overall treatment time. The applied protocol revealed a good reproducibility in fiducial repositioning between different fractions, as demonstrated by the obtained *RE_FB* of fiducials registration, which measured 2.1 ± 1.6 mm (Table 2). No side effects associated to the daily sticking of surface fiducials, such as local worsening of the skin erythema or infection, were observed in the analyzed patients. Moreover, the applied fiducials did not generate any artifact visible in the CT simulation scan.

## 5 CONCLUSIONS

The use of multiple surface fiducials monitored through 3D optical tracking systems was investigated for the control of left‐breast DIBH radiotherapy. Improved results in surface and target reproducibility were found with the proposed fiducial‐based approach, which allows a more robust compensation of interfraction variations in patients' breathing baseline, while providing a quantitative indication of residual uncertainties linked to possible marker repositioning uncertainties and to the deformable component of patient setup error. Future works will be focused on the evaluation of the dosimetric consequences within target and organs at risk associated to the DIBH uncertainties under RPM‐based control, compared to the proposed fiducial‐based approach, as this is the only way to prove whether the effort of a more complex approach to DIBH monitoring may lead to significant improvement in normal tissue sparing and target coverage.

## CONFLICT OF INTEREST

No conflicts of interest.
